# Pediatric combined hepatocellular-cholangiocarcinoma (cHCC-CC) with neuroendocrine features: distinguishing genetic alterations detected by chromosomal microarray

**DOI:** 10.1186/s13000-023-01305-z

**Published:** 2023-02-13

**Authors:** Alyeesha B. Wilhelm, Arwyn G. Cunningham, Cynthia Kassab, Eric C. Fitz, Jianli Dong, Ravi S. Radhakrishnan, Sarangarajan Ranganathan, Dongfeng Tan, Heather L. Stevenson

**Affiliations:** 1 Department of Pathology, The University of Texas Medical Branch, Galveston, TX USA; 2 Department of Surgery, The University of Texas Medical Branch, Galveston, TX USA; 3 Department of Pathology, Cincinnati Children’s Hospital, Cincinnati, OH USA; 4 Department of Pathology, M.D. Anderson Cancer Center, Houston, TX USA

**Keywords:** cHCC-CC, Cholangiocarcinoma, Hepatoblastoma, Hepatocellular Carcinoma, Liver, Neuroendocrine, Pediatric

## Abstract

**Background:**

Liver tumors exhibiting hepatocellular, cholangiocarcinoma, and neuroendocrine features are extremely rare, with only five cases reported in the literature.

**Case presentation:**

We present an unusual case of a combined hepatocellular-cholangiocarcinoma (cHCC-CC) with neuroendocrine features in a pediatric patient. A 16-year-old presented with abdominal pain and a 21.0 cm mass in the right hepatic lobe with extension into the left lobe. Histology showed a poorly differentiated tumor with a solid, tubuloglandular, and microcystic architecture. Immunohistochemistry results were negative for hepatic markers, positive for markers of biliary differentiation, and positive for neuroendocrine differentiation. The neoplasm was reviewed at several institutions with differing diagnoses. Single nucleotide polymorphism (SNP) chromosomal microarray (CMA) showed large deletions within chromosomes 6q and 13q in both the hepatocellular-like areas and the cholangiocarcinoma-like areas, with additional large deletions in the cholangiocarcinoma-like areas, supporting origin from hepatocellular carcinoma. The final diagnosis was a cHCC-CC with neuroendocrine features.

**Conclusions:**

Diagnosis of cHCC-CCs relies predominately on histomorphology, as per the 2018 International Consensus Group on the nomenclature of cHCC-CC. These findings in this case support that the pathological classification of these lesions be based on molecular data, which could better direct treatment. Further classification of cHCC-CCs and determination of their clinicopathological relevance will require more interobserver consistency and continued molecular profiling of these lesions.

## Background

Primary pediatric liver tumors are uncommon, with the vast majority being hepatoblastomas, followed by conventional hepatocellular carcinomas (HCC). Molecular studies have shown that the vast majority of hepatoblastomas have mutations in the Wnt/β-catenin pathway, whereas HCCs show extensive variation in genetic mutations. Tumors with biliary and neuroendocrine (NE) features have been primarily described in adult patients, with only rare incidence in children. These include combined hepatocellular-cholangiocarcinoma (cHCC-CC) and mixed neuroendocrine–non-neuroendocrine neoplasm (MiNEN) [1–3]. Molecular analysis may be used to determine tumor cell lineage, facilitating proper diagnosis and treatment.

## Case presentation

A 16-year-old, obese (BMI 37), Hispanic man with no significant past medical history presented to the emergency department with sharp, persistent epigastric pain. Family history was negative for any known cancer or chronic gastrointestinal illnesses. Physical examination was unremarkable. Laboratory tests were negative for hepatitis C (HCV), showed immunity to hepatitis B (HBV), and showed mildly elevated liver transaminase levels.

**Fig. 1:**
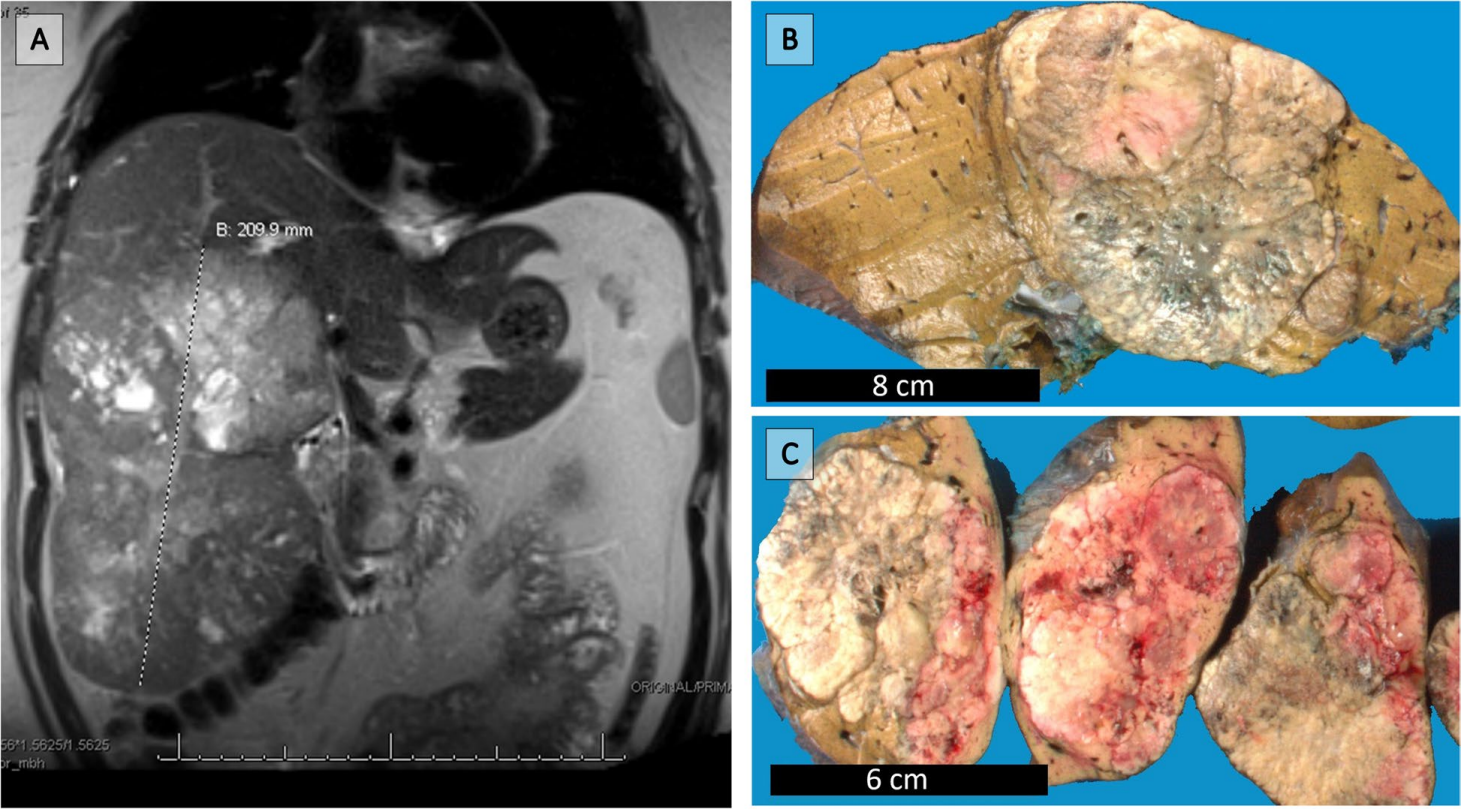
T2 MRI shows an enlarged liver measuring 29.0 × 22.0 x 10.0 cm with mass lesion (**A**). Grossly identified are two tan-white to tan-pink, focally hemorrhagic abutting tumors measuring 21.5 cm and 4.5 cm (**B** and **C**)

Ultrasonography (US) revealed a partially visualized heterogeneous mass with irregular lobulated borders. Computed tomography (CT) and magnetic resonance imaging (MRI) of the abdomen and pelvis revealed a 21.0 cm partially necrotic mass occupying most of the right hepatic lobe (Fig. 1A). Subsequent CT thorax and whole-body bone scan showed no other lesions or evidence of metastatic disease. The serum alpha-fetoprotein level was within normal limits. A right extended hepatectomy was performed, which was complicated by blood loss requiring a massive transfusion protocol.

## Methods

### Immunohistochemistry

Immunohistochemistry (IHC) was performed on paraffin-embedded tissue using standard techniques for β-catenin (Dako/RTU; monoclonal mouse, clone β-catenin), alpha-fetoprotein (Dako/RTU; polyclonal rabbit), Hep-Par1 (Dako/RTU; monoclonal mouse, clone OCH1Eg), glypican-3 (Ventana/RTU, monoclonal mouse, clone GC33), pCEA (Cell Marque/RTU; polyclonal rabbit), CK7 (Ventana/RTU; polyclonal rabbit, clone SP52), CK19 (Dako/RTU; monoclonal mouse, clone RCK108), chromogranin A (Ventana/RTU; clone LK2H10), and synaptophysin (Ventana/RTU; monoclonal rabbit, clone SP11). Staining using Fontana-Masson (manual stain), periodic acid-Schiff after diastase (PAS-D) (Ventana kit), iron (Ventana kit), and copper (manual stain) was also performed. Additional staining methods from outside institutions during consultation are not described here.

### Chromosomal microarray analysis

Chromosomal analysis was performed by extracting and purifying the patient’s genomic DNA (gDNA) from tissue samples using a QIAamp DNA FFPE Tissue Kit (Qiagen Inc., Valencia, CA, USA). A chromosomal microarray (CMA) was conducted to identify copy number variations (CNV) using the OncoScan CNV Assay Kit (Affymetrix, Santa Clara, CA, USA). A CNV profile was generated and analyzed using the Chromosomal Analysis Suite (Affymetrix, Santa Clara, CA, USA).

### Next generation sequencing

Next generation sequencing (NGS) analysis was performed at M.D. Anderson Cancer Center on DNA extracted from tissue sample in their CLIA-certified molecular diagnostics laboratory. PCR-based sequencing was performed using a NGS platform on genomic DNA to screen for somatic mutations in the coding sequence of 134 genes and selected copy number variations in 47 genes. Total genes tested was 146 (overlap) and included *RB1*, *TP53*, and *ARID1A*. NGS sequencing analysis of these genes was further confirmed by other platforms during validation. The genomic reference sequence used was GRCh37/hg19. Also performed was NGS-analysis for fusion sequences. Copy DNA prepared from extracted RNA was combined with targeted amplicon based NGS to amplify both a set of expected control RNA sequences and a set of targeted fusion sequences corresponding to clinically relevant known inter- and intragenic fusions in 51 genes. Sequences were aligned against a synthetic fusion genome to identify fusions by coverage analysis. A post-variant calling analysis and annotation tool, OncoSeek version 1.10.1.532, was used in the construction of the report.

## Results

### Pathology and IHC

The pathology department received a partial hepatectomy measuring 29.0 cm with an intact capsule. Sectioning revealed two tan-white to tan-pink, focally hemorrhagic abutting tumors measuring 21.5 cm and 4.5 cm respectively (Fig. 1B-C). The uninvolved hepatic parenchyma was tan-yellow and grossly unremarkable.

**Fig. 2:**
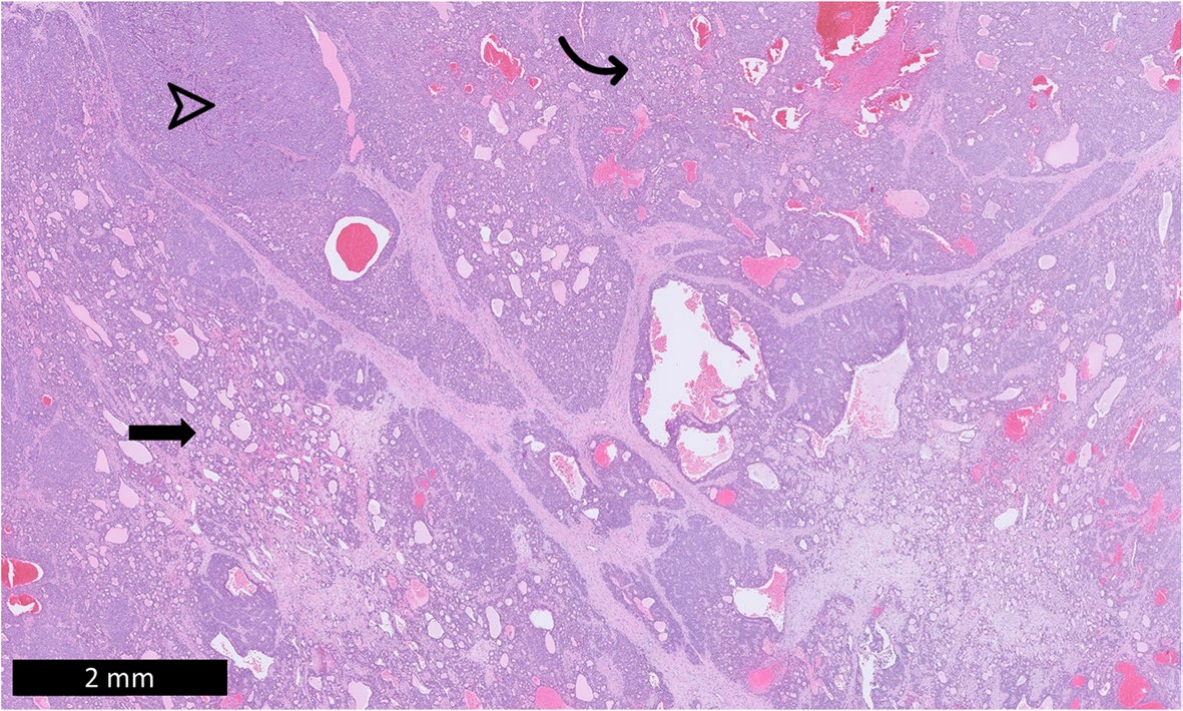
The mass demonstrates multiple histologic patterns including tubuloglandular structures (curved arrow), solid sheets and trabeculae (arrowhead), and small spaces lined by biliary-like epithelium and filled with eosinophilic proteinaceous luminal material (arrow). (H&E)

**Fig. 3:**
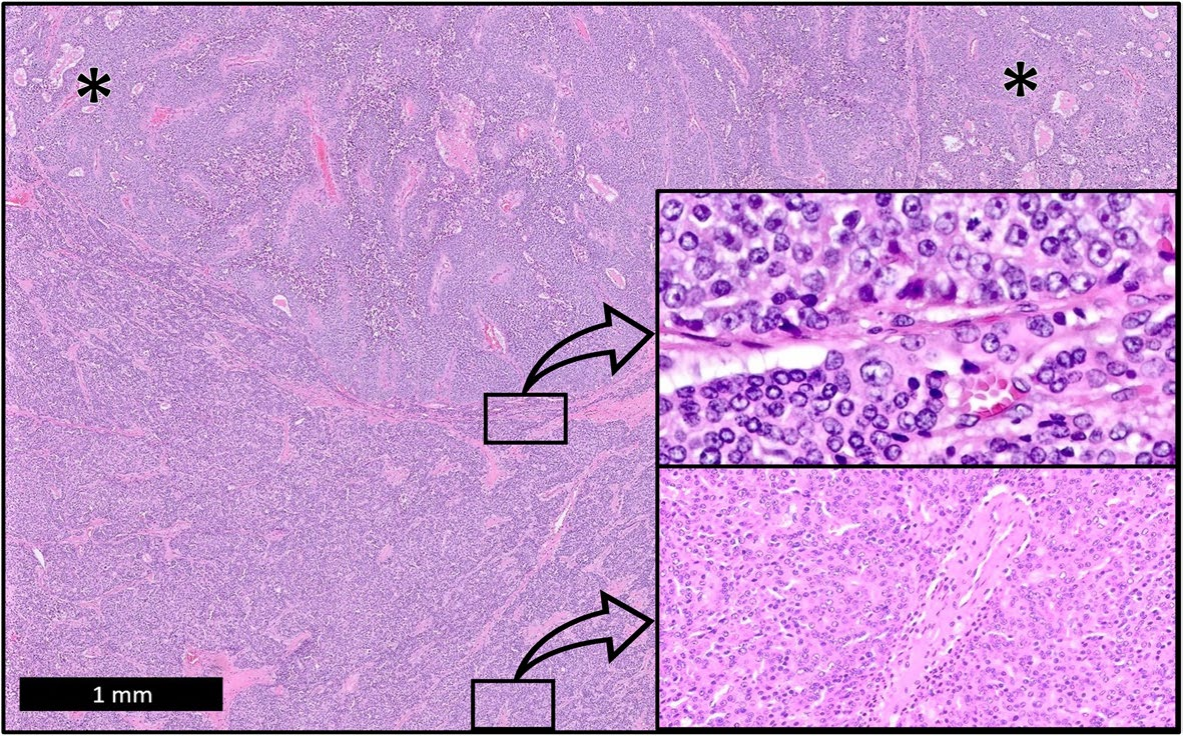
Two distinct histological patterns of the tumor are identified, with cholangiocytic differentiation (asterisks) identified in the top lesion (H&E, low power). Small uniform tumor “cancer stem cells” with small basophilic vesicular nuclei, occasional inconspicuous nucleoli, scant pale eosinophilic cytoplasm, indistinct cell borders, and frequent mitoses are seen throughout and are prominent at transitional zones (top inlay; H&E, high power). Tumor cells of similar cytology with more abundant eosinophilic cytoplasm surround an unpaired artery in the HCC-like areas (bottom inlay; H&E, intermediate power)

Histological sections from the liver tumors were reviewed by three board-certified liver/GI and pediatric pathologists, H.L. Stevenson, S. Ranganathan, and D. Tan. Microscopy revealed a large heterogeneous mass surrounded by a thick fibrous capsule with vascular channels. The mass demonstrated multiple histological patterns, including solid sheets, trabeculae, and tubuloglandular structures, as well as spaces lined by biliary-like epithelium (Fig. 2). Most neoplastic cells exhibited stem cell-like features [1] with small vesicular nuclei, inconspicuous nucleoli, pale eosinophilic cytoplasm, and indistinct cell borders (Fig. 3). Tumor cells in the solid and trabecular areas surrounded unpaired arteries and were slightly larger with more pale eosinophilic cytoplasm and focally prominent nucleoli (Fig. 3); these tumor nests were focally interrupted by frequent transgressing vessels. These features are more consistent with hepatocellular differentiation. Mitoses were frequent (up to 5–6 per high power field). No frank bile production was observed. The non-neoplastic liver was non-cirrhotic, showed minimal portal inflammation, and macrovesicular steatosis involved approximately 30% of hepatocytes. No viral inclusions or confluent necrosis were observed.

**Fig. 4:**
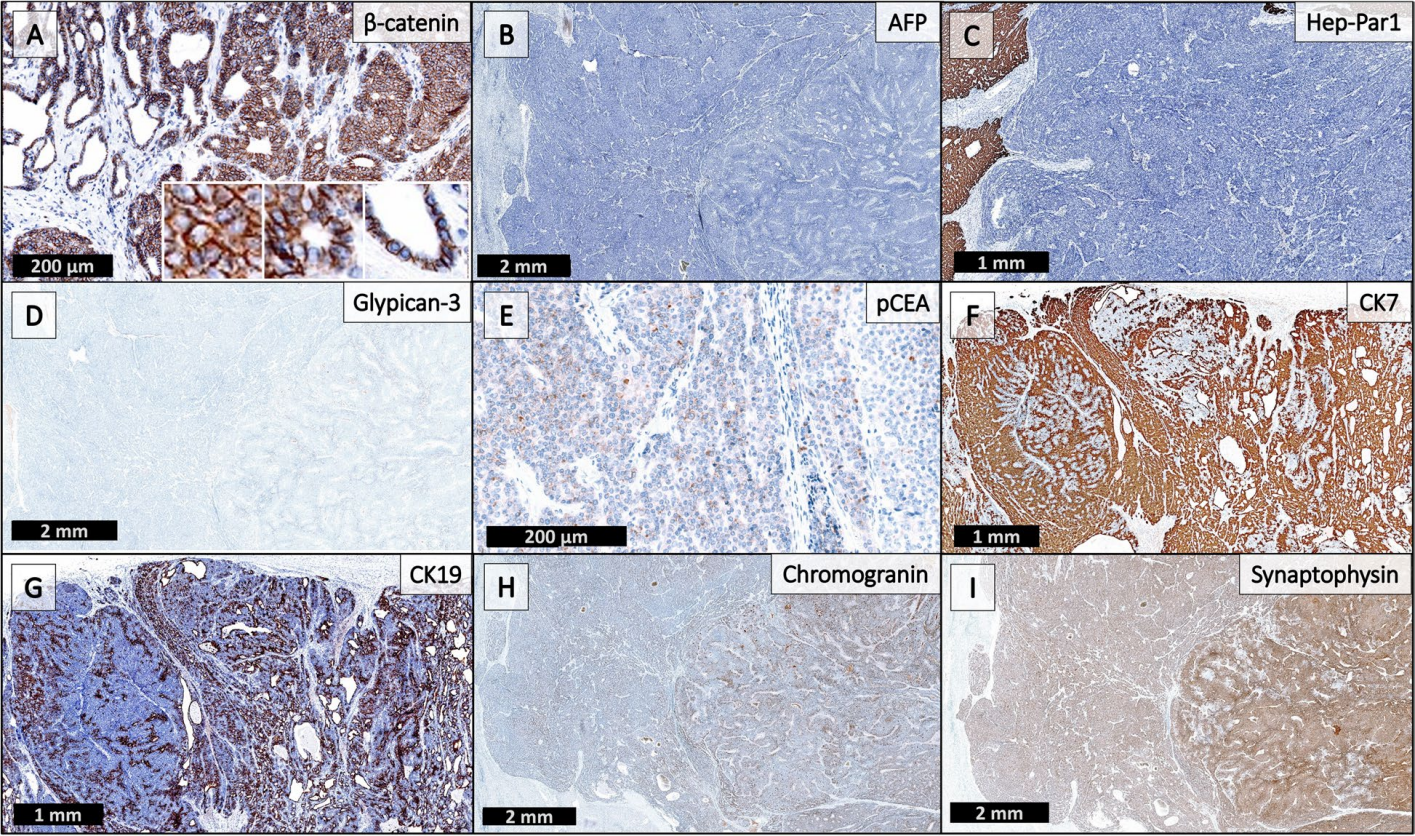
IHC panel. β-catenin (**A**) shows membranous but not nuclear staining, while alpha-fetoprotein (**B**) is negative; these findings are not supportive of hepatoblastoma. Negative Hep-Par1 (**C**), negative Glypican-3 (**D**), and largely non-canalicular staining pattern of pCEA (**E**) suggest poorly differentiated tumor if of hepatic origin. CK7 (**F**) and CK19 (**G**) show positive staining (strongly positive within the ductular components), highlighting cholangiolar-like differentiation. Focal areas of strongly positive chromogranin (**H**) and diffusely positive synaptophysin (**I**) highlight neuroendocrine-like differentiation

A large battery of immunohistochemical stains (IHC) (Fig. 4) was performed at three separate institutions (Table 1). The neoplasm was positive for CK7, CK19, and synaptophysin; weakly positive for pCEA and chromogranin; and negative for nuclear β-catenin, Hep-Par1, glypican-3, alpha-fetoprotein, and glutamine synthetase. Across the institutions, the two diagnoses established were a combined hepatocellular-cholangiocarcinoma (cHCC-CC) and an epithelial tumor with neuroendocrine differentiation.

**Table 1:** Immunohistochemical Stains Performed

Immunohistochemical stain performed	Results
Alpha-fetoprotein	Negative
β-Catenin	Negative nuclear staining. Membranous staining diffusely positive in all components
Hep-Par1	Negative
Glypican-3	Negative
Glutamine Synthetase	Patchy single cell staining in some solid areas only; negative in the glandular structures
pCEA	Focal canalicular positivity
CK7	Positive and highlights biliary-like profiles
CK19	Positive and highlights biliary-like profiles
Pankeratin	Positive. Negative in a subset of the tumor
BCL-2	Focal weak positivity
Cyclin-D1	Focal weak positivity
CD99	Negative
Synaptophysin	Positive. Some positivity within the biliary-type cells
Chromogranin	Negative — Focal strong positive (institution variable)
Ki-67 (Computer Image Analyzer)	Positive: 17.95% (hotspot); 14.15% (average)
SALL-4	Negative
Arginase	Negative
PROX-1	Negative
p53	Negative
MOC-31	Strong positive; cholangiolar component stronger than hepatocellular component
CD56	Negative
CAM 5.2	Positive in the glandular (CC-like) elements. Negative in the solid (HCC-like) areas
CA 19.9	Focally positive
MSH6	Intact nuclear staining
PMS2	Intact nuclear staining
TTF-1	Negative
OCT 3/4	Negative

Stains were performed across three different institutions and are listed in the order in which they were ordered. Results were consistent across institutions unless otherwise specified

Staining results refer to the tumor cells unless otherwise specified

### Molecular

**Fig. 5:**
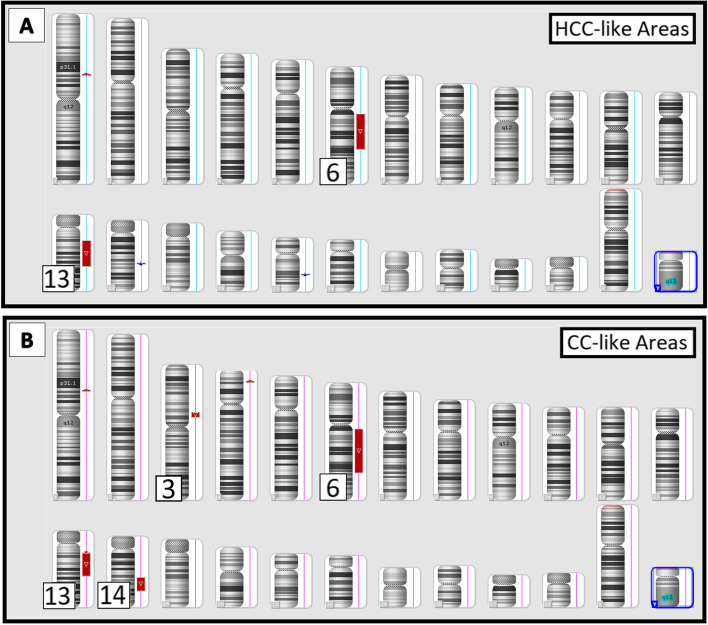
Chromosomal microarray (CMA) was performed on the histologically HCC-like (**A**) and histologically CC-like (**B**) areas. Both areas revealed large deletions (red bars) in chromosomes 6q and 13q and the CC-like component revealed additional large deletions in chromosomes 3p and 14q. The results favor that the neoplastic cells arose from a single clone with genetic heterogeneity causing the tumor’s histologic diversity

Molecular tests for *BRAF* and *KRAS* mutations (mutations common to CC) were performed and were negative. Given the unusual histomorphology and differing diagnoses, CMA was performed on both the HCC-like and CC-like areas (Fig. 5), as well as on the patient’s benign liver tissue. In both the HCC-like and CC-like components, large deletions (> 5 Mb) were identified in chromosomes 6q and 13q. These deletions are known to be common in HCCs [4–7]. Additional large deletions (> 5 Mb) were identified in chromosomes 3p and 14q in the CC-like component. It is favored that the neoplastic cells arose from a single clone with genetic heterogeneity and a subclone resulted in the CC-like histologic diversity. All copy number variations were absent in the patient’s normal liver, supporting their somatic origin.

#### Final diagnosis and follow-up

Based on the immunohistomorphology and review of the multidisciplinary 2018 International Consensus on the nomenclature of cHCC-CC, the final diagnosis was concluded to be a cHCC-CC with neuroendocrine features. Chromosomal analysis indicated that the neoplastic cells arose from an HCC.

At the patient’s six-month follow-up appointment, a CT scan showed new bilateral lung masses and new masses in his remaining liver. Liver core needle biopsy showed a tumor morphologically similar to the previous resection with frequent mitotic figures. (31 per 2 mm^2^), frequent apoptotic bodies, and no necrosis. Staining for NE markers, HepPar1, Arginase 1, and Glypican 3 produced results similar to that of the previous resection specimen. The findings confirmed the recurrence of the original neoplasm. Next-generation sequencing (NGS) was performed on the biopsy specimen and revealed a mutation of *FANCI* (non-specific). No gene fusions were identified.

## Discussion and conclusions

Differential diagnoses for this case initially included hepatoblastoma, primary hepatic neuroendocrine neoplasm, and combined hepatocellular-cholangiocarcinoma (cHCC-CC).

Hepatoblastomas are the most common primary hepatic tumors in the pediatric population, most commonly affecting children less than 5 years-of-age [1, 2, 8]. The vast majority have mutations in the Wnt/β-catenin pathway, reflected in a positive nuclear β-catenin by IHC. A diagnosis of ‘hepatoblastoma with cholangioblastic features’ was given serious consideration prior to the completion of IHC studies. Histology focally resembled a hepatoblastoma of epithelial subtypes (fetal and macrotrabecular) as described by Auld et al*.* However, IHC was negative for nuclear beta-catenin, there was minimal staining for glutamine synthetase and cyclin-D1, both IHC and serum were negative for AFP, and the lesion contained no genetic markers known to hepatoblastoma [9]. Furthermore, stains for CK7 and CK19 were more supportive of at least focal biliary differentiation.

Primary hepatic neuroendocrine tumors are exceedingly rare, requiring a thorough evaluation to rule out metastasis from other sites [10, 11]. Their diagnosis is controversial because of the scarcity of neuroendocrine cells present within the normal liver. Few studies have identified mutations in primary hepatic neuroendocrine tumors; however, one study identified mutations in TP53, similar to poorly differentiated tumors of other organs [12]. The diagnosis of a NE neoplasm is often considered in the differential for a cHCC-CC, as evidenced in this case by the cytologic features and the variable but diffuse staining for synaptophysin and chromogranin. Cytology was diffusely reminiscent of a NE carcinoma and IHC staining for synaptophysin and chromogranin was variable throughout both tumor morphologies (Fig. 3, top inlay & Fig. 4). A mixed neuroendocrine–non-neuroendocrine neoplasm (MiNEN) was also considered, however, a distinct NE and non-NE component could not be discerned by morphology or IHC. The prominent HCC-like architecture, HCC-like chromosomal findings, and CC-like biliary structures with CC-like IHC patterns, in this case, were most supportive of a cHCC-CC. The epithelial components, in this case, were non-supportive of a primary hepatic neuroendocrine tumor.

Diagnosis of an HCC with CC-like and NE-like features was considered after CMA, which showed mutations common to HCCs in both the HCC-like and the CC-like areas and favored origin from a single cell line. The 2018 International Consensus Group on the nomenclature of cHCC-CC, however, recommends that the diagnosis of cHCC-CC be determined on routine stains (e.g., H&E), as IHC provides only supplemental evidence [13]. Therefore, at present, this lesion is best diagnosed as a cHCC-CC with neuroendocrine features. Authors of the 2018 consensus do, however, comment on the importance of molecular studies in these lesions and they highlight the significant heterogeneity observed [13]. The CMA results from this case support a single clonal process. Furthermore, the additional deletions unique to the CC-like areas indicate a clonal divergence, giving rise to the genetic heterogeneity within the tumor and the corresponding histologic diversity. These findings have been previously demonstrated in larger studies [5, 14]. Moeini et al*.* revealed distinct molecular profiles for the entities described under the umbrella of cHCC-CC and recommend that the pathological classification of cHCC-CCs be redefined based on new molecular data [14]. Based on our findings, this is a reasonable consideration.

A review of the literature showed only five other cases with hepatoid, biliary, and neuroendocrine features in primary liver tumors (Table 2). Each of the previous cases is unlike that described here. Beard et al*.* described a neoplasm of similar morphology in a young adult, although with focal Hep-Par1 staining and distinct molecular findings which suggest the various components arose from distinct cell lineages [15]. Braxton et al*.* described three cases – two in young patients – with strikingly similar morphology and IHC to our case as well as a common loss in chromosome 6q. The remaining chromosomal abnormalities, however, classify these lesions as cholangiocarcinomas rather than HCCs [16]. Dimopoulos et al*.* described a case diagnosed as HCC with biliary and neuroendocrine differentiation, although in an elderly woman with a history of HCV who had positive serum and IHC for AFP, and positive IHC for Hep-Par1 [17]. We found no previously described cases of cHCC-CC with NE-like features involving a patient within the pediatric age group. Furthermore, no previous cases with this diagnosis have presented with negative serum tumor markers, negative IHC common in hepatocyte differentiation, and this unique cytogenomic profile.

**Table 2:** Hepatic carcinomas reported with similar morphology (HCC, CC, and NE)

**Reference**	**Age/Sex**	**Morphology**	**Serum Markers**	**IHC (+) **	**IHC (–)**	**Molecular**	**Diagnosis**
Beard et al. [11]	19/M	Tubulocystic, trabecular, glandular	CA 19–9	CA19.9, CK19, CK7, Hep-Par1 (few cells), Arginase-1, Syn	NA	Copy number imbalances (e.g., 5q and 7p). NE mutations not identified in the HCC/CC components indicating separate cell line lineages	Malignant epithelial neoplasm with multiple lineages including HCC, CC, and NE carcinoma
		^a^Nodular/solid		^a^CK19 (focal), Hep-Par1 (patchy), Syn, CD56	^a^CK7		
Braxton et al. [12]	17/F	Microcystic, trabeculae, pseudoglandular, solid/hepatoid, blastemal	None	CK19, CK7, Syn & CgA (weak), InhibinA	Hep-Par1, AFP, b-Catenin	Cytogenomic alterations consistent with primary liver carcinomas (not NE carcinomas)	Cholangioblastic Cholangiocarcinoma
	44/F	Trabecular, solid/hepatoid	CgA (mild)	CK7 (weak), CK19, pCEA (non-canalicular), AFP (weak, patchy), Glypican-3, CDX2 (focal), Syn & CgA (weak), InhibinA	Ca19.9, Hep-Par1		
	24/F	Microcystic, trabeculae, pseudoglandular, solid/hepatoid, blastemal	None	CK7, CK19, SOX9, pCEA (non-canalicular), CgA (weak), InhibinA	b-Catenin, Glypican-3, Syn		
Dimopoulos et al. [13]	65/F	Tubuloglandular, solid, nested	AFP AFP-L3% CgA	CK19 and focally ( +) for CK7, CK20, AFP, Hep-Par1, Arginase 1, pCEA, Syn, NSE, CDX2, p53	CA19.9, CgA	NGS: Mutations in CDKN2A exon 2 and TP53 exon 7	HCC with biliary and NE differentiation
				^a^Syn (strong, diffuse), CgA (focal, weak)	^a^AFP, Hep-Par1, Arginase 1, CD56		
This case	16/M	Tubuloglandular, biliary-like, trabecular, solid	None	CA19.9 (focal), CK19 (biliary areas), pCEA (non-canalicular, focal), Syn & CgA (weak)	AFP, b-catenin, Hep-Par1, Glypican 3	CMA: HCC-like and CC-like areas share common LOH pattern with additional losses detected in the CC-like areas. Findings indicate same cell line lineage with subsequent genetic diversion giving rise to CC-like phenotype	cHCC-CC with NE-like features

*AFP* Alpha-fetoprotein, *CC* Cholangiocarcinoma, *CgA* Chromogranin, *CMA* Chromosomal microarray, *HCC* Hepatocellular carcinoma, *IHC* Immunohistochemistry, *LOH* Loss of heterozygosity, *NA* Not applicable, *NE* Neuroendocrine, *NGS* Next generation sequencing, *Syn* Synaptophysin

^a^Features describe a subset or a metastatic lesion of the primary tumor

Primary pediatric HCCs must be considered in the differential for tumors such as this one, especially since hepatocellular components may be positive for CK7 and CK19 [1]. Pediatric risk factors for HCC include prenatal HBV, cholestatic disorders (e.g., biliary atresia, familial cholestasis), and metabolic diseases, all absent in our patient [1, 18, 19]. HCCs may accumulate many genetic variants during tumor initiation and progression, especially those arising in a background of cirrhosis. Common mutations observed in HCCs include mutations in the TERT promoter, TP53, Wnt/β-catenin pathway, and loss of heterozygosity of different chromosomes [8].

HCCs with CC-like components tend to have a poor prognosis, with an increased risk of recurrence and metastasis, and an overall poor survival rate. One study reported one- and five-year survival rates of 41.9% and 17.7%, respectively, with a median survival of 8 months [20]. Outcomes improved with the combination of surgery, radiation, and chemotherapy.

Combined HCC-CCs are extremely rare in the pediatric population, and this case demonstrates that they may also be extremely difficult to diagnose using both histomorphology and IHC. We recommend consultation, review of the present consensus terminology, and molecular genetic analysis to aid in the diagnosis and molecular categorization of these peculiar, uncommon, and poorly differentiated tumors.

## Data Availability

Data sharing is not applicable to the article as no datasets were generated or analyzed during the current study.

## Abbreviations

BMI: Body mass index
CC: Cholangiocarcinoma
cHCC-CC: Combined hepatocellular-cholangiocarcinoma
CMA: Chromosomal microarray
CNV: Copy number variations
CT: Computed tomography
HBV: Hepatitis B virus
HCC: Hepatocellular carcinoma
HCV: Hepatitis C virus
IHC: Immunohistochemistry
MiNEN: Mixed neuroendocrine–non-neuroendocrine neoplasm
MRI: Magnetic resonance imaging
NE: Neuroendocrine
SNP: Single nucleotide polymorphism
